# Clinical outcomes of needle-perc-assisted endoscopic surgery for 2–4 cm renal calculi: a propensity score-matched comparative study with subgroup analysis by stone infection status

**DOI:** 10.3389/fmed.2026.1938484

**Published:** 2026-10-22

**Authors:** Yuxian Wang, Peizhi Zhang, Zheng Xu, Daxun Luo, Yuzhe Tang, Jianxing Li

**Affiliations:** Department of Urology, Beijing Tsinghua Changgung Hospital, School of Clinical Medicine, Tsinghua Medicine, Tsinghua University, Beijing, China

**Keywords:** needle-perc-assisted surgery, nephrolithiasis, percutaneous nephrolithotomy, propensity score matching, retrograde intrarenal surgery

## Abstract

**Purpose:**

To compare needle-perc-assisted PCNL (NS) and needle-perc-assisted RIRS (NR) with Standard PCNL for 2–4 cm renal calculi and explore effect modification by infectious-stone status.

**Patients and methods:**

This retrospective cohort included 365 patients treated between January 2021 and September 2025. Two independent 1:1 propensity score-matched comparisons were constructed. The primary outcome was the 1-month stone-free rate (SFR), defined as no residual fragment ≥4 mm on KUB radiography. Matched outcomes were analyzed using paired methods, with Holm adjustment for the two principal SFR comparisons.

**Results:**

Matching yielded 48 Standard PCNL–NS pairs and 86 Standard PCNL–NR pairs. SFR was 85.4% with NS vs. 72.9% with Standard PCNL (RD, 12.5%; 95% CI, −3.6% to 28.6%; exact McNemar *P* = 0.210; Holm-adjusted *P* = 0.210) and 91.9% with NR vs. 80.2% with Standard PCNL (RD, 11.6%; conditional exact 95% CI, −0.1% to 20.1%; exact McNemar *P* = 0.052; Holm-adjusted *P* = 0.104). Exploratory analyses associated NS with less fever and a smaller postoperative WBC increase but greater analgesic use. NR was associated with less analgesic use, longer operative time, and shorter hospitalization. Treatment-by-infectious-stone-status interactions were not statistically significant for NS or NR (*P* = 0.647 and *P* = 0.942).

**Conclusion:**

SFR estimates numerically favored NS and NR, but superiority over Standard PCNL was not established. These exploratory findings support further prospective evaluation of Needle-perc-assisted surgery in selected patients.

## 1 Introduction

Urolithiasis is one of the most common diseases of the urinary system, and its global prevalence continues to increase. Between 2000 and 2021, the number of newly diagnosed cases increased by 26.7% worldwide (1, 2). For renal calculi >2 cm, percutaneous nephrolithotomy (PCNL) remains the guideline-recommended first-line treatment (3), but its tract may contribute to bleeding, renal injury, and infectious complications (4). Needle-perc is a 4.2-Fr percutaneous nephroscope designed to provide targeted access to unfavorable calyces and residual stones with minimal additional tract injury (5).

Advances in flexible ureteroscopy and laser lithotripsy have expanded retrograde intrarenal surgery (RIRS) to selected stones >2 cm (3, 6, 7). Nevertheless, high stone burden, lower-pole anatomy, narrow infundibula, and calyceal diverticula may limit access and stone clearance.

Infectious stones are associated with perioperative infection, renal deterioration, and recurrence. Bacteria embedded within the stone matrix may persist despite antibiotics and contribute to postoperative events (8, 9). Evidence regarding needle-perc-assisted surgery in this population remains limited.

We therefore used propensity score matching (PSM) to compare needle-perc-assisted PCNL (NS) and needle-perc-assisted RIRS (NR), a modified ECIRS approach combining Needle-perc with flexible ureteroscopy, with Standard PCNL for 2–4 cm renal calculi, including analyses by infectious-stone status.

## 2 Materials and methods

### 2.1 Study design and participants

This single-center retrospective cohort included patients treated with Standard PCNL, needle-perc-assisted PCNL (NS), or needle-perc-assisted RIRS (NR) for 2–4 cm renal calculi at Beijing Tsinghua Changgung Hospital between January 2021 and September 2025.

Eligible patients had renal calculi diagnosed by non-contrast computed tomography, a cumulative stone diameter of 2–4 cm, treatment with one of the three study procedures, and the required clinical and imaging data. Exclusion criteria were ipsilateral urinary tract malignancy, severe hepatic or renal insufficiency, systemic coagulation disorders, severe ipsilateral renal atrophy, uncontrolled urinary tract infection, salvage conversion, and pure uric acid or L-cystine stones. Of 490 patients assessed, 125 were excluded. The 32 radiolucent or faintly radiopaque stones excluded because SFR was evaluated by KUB comprised 21 Standard PCNL, three NS, and eight NR cases.

The initial surgical approach was selected by a senior surgeon in the treating team according to routine guideline-based clinical practice and preoperative clinical and anatomical assessment. No study-specific standardized treatment-allocation algorithm was used. The intended procedure and detailed procedure-specific selection criteria were not recorded as dedicated variables. The intended approach was inferred from operative records when possible, but the rationale for selecting Standard PCNL, NS, or NR in individual patients could not be reliably reconstructed. Salvage conversion was defined as failure to complete the initially intended retrograde ureteroscopic strategy, requiring intraoperative conversion to a percutaneous procedure, rather than the planned or criterion-based addition of Needle-perc within an NS or NR procedure. Operative records indicated that all nine excluded salvage conversions were initially intended to undergo a retrograde ureteroscopic procedure but were converted to Standard PCNL or NS because ureteral conditions prevented successful ureteroscope advancement. These cases were excluded because the unsuccessful retrograde attempt introduced additional operative time that could not be attributed to the definitive procedure.

The final cohort comprised 365 patients: Standard PCNL (*n* = 186), NS (*n* = 79), and NR (*n* = 100). This retrospective study was approved by the Ethics Committee of Beijing Tsinghua Changgung Hospital (No. 24755-0-02), which waived the requirement for informed consent. The study complied with the Declaration of Helsinki and institutional policies.

### 2.2 Preoperative preparation

Preoperative evaluation included blood count, urinalysis, urine culture, hepatic and renal function, coagulation testing, chest radiography, electrocardiography, renal ultrasonography, KUB radiography, and non-contrast CT. Two experienced radiologists or urologists independently assessed stone count, total diameter, density, and parallel calices on PACS. Maximum diameters were measured on the largest axial section and summed for multiple stones. Density was the mean of three central measurements obtained with a circular region of interest approximately one-third of the stone diameter, avoiding margins and heterogeneous calcification. Parallel calices were adjacent calyces that could not be accessed through the primary tract, or could be accessed only by marked tract angulation with potential parenchymal distortion or injury. Interobserver agreement was not formally quantified.

The CT urography-based B.T.C.H. score comprised stone burden (B), renal-pelvis type (T), calyces involved (C), and hydronephrosis (H). Scores were: B, solitary/multiple/partial staghorn/complete staghorn = 1/2/3/4; T, single/divided pelvis = 1/3; C, 1–2/3–4/≥5/indeterminate because of abnormal anatomy = 1/2/3/4; and H, mild/severe/localized due to infundibular obstruction/none = 1/2/3/4. Total scores of 4–8, 9–12, and 13–15 defined grades I–III (10). Guy's stone score (GSS) grades were: I, a solitary pelvic or middle/lower-calyceal stone in normal anatomy; II, a solitary upper-pole stone, multiple stones in normal anatomy, or a solitary stone in abnormal anatomy; III, multiple stones in abnormal anatomy, a calyceal-diverticular stone, or partial staghorn calculus; and IV, complete staghorn calculus or stones in patients with spina bifida or spinal cord injury (11).

Preoperative urinalysis and midstream urine culture were routinely performed. When empirical antimicrobial treatment was indicated, a second-generation cephalosporin or levofloxacin was generally administered and subsequently adjusted according to urine-culture and antimicrobial-susceptibility results when available. Definitive stone surgery was deferred until fever and other clinical evidence of uncontrolled infection had resolved. Patients with infected urinary obstruction underwent drainage using a ureteral stent or percutaneous nephrostomy when clinically indicated. Detailed antibiotic durations and drainage data were not recorded consistently in the retrospective database. Renal pelvic urine and stone cultures were not routinely obtained during the study period.

### 2.3 Surgical procedures

#### 2.3.1 Standard PCNL group

Under general anesthesia, a 5-Fr ureteral catheter was placed in lithotomy position for saline irrigation, after which patients were positioned prone or, when necessary, lateral. The target calyx was punctured under real-time ultrasonography according to the 30-degree principle (12) using a 17.5-G needle. After ultrasound-guided guidewire placement, a 24-Fr tract was created with Alken coaxial or high-pressure balloon dilators. Stones were treated with combined ultrasonic/pneumatic lithotripsy. A 6-Fr ureteral stent, nephrostomy tube, and Foley catheter were routinely placed.

#### 2.3.2 Needle-perc-assisted PCNL (NS) group

NS comprised one or more 24-Fr PCNL tracts supplemented by an undilated 4.2-Fr Needle-perc tract. After most stone burden had been removed, residual stones inaccessible through the standard tract were localized by ultrasonography and nephroscopy. Needle-perc was advanced under real-time ultrasound guidance, with endoscopic confirmation of calyceal entry and stone visualization. Stones were fragmented using a 200 μm holmium laser fiber at 12–20 W. Larger fragments were pushed under direct vision with the Needle-perc tip or mobilized toward the main tract by irrigation at 500 mmHg and 800 mL/min. A ureteral stent, nephrostomy tube, and Foley catheter were routinely placed.

#### 2.3.3 Needle-perc-assisted RIRS (NR) group

Patients underwent NR under general anesthesia in the Galdakao-modified supine Valdivia position. Two surgeons operated simultaneously: one performed flexible ureteroscopy and the other established ultrasound-guided Needle-perc access. After access-sheath placement, the collecting system was examined with an 8.5-Fr flexible ureteroscope. Needle-perc was added only when ureteroscopic exploration confirmed that RIRS alone could not reach, relocate, or effectively treat the target stone.

Needle-perc puncture was performed without fluoroscopy under real-time ultrasound guidance, with visual confirmation of calyceal entry and the target stone. Stones were fragmented with a 200 μm holmium laser fiber. Fragments were pushed with the Needle-perc tip or irrigated at 500 mmHg and 800 mL/min toward the renal pelvis, a larger calyx, or the extraction route, then treated through either endoscope. Small fragments were basketed and dust evacuated through the access sheath. Irrigation was supplied by gravity, a single-action pump, or, in a spacious calyx, a continuous pump. Needle-perc was removed without nephrostomy placement; a 6-Fr ureteral stent and Foley catheter were routinely placed.

### 2.4 Stone composition analysis

Postoperative stones were analyzed by infrared spectroscopy. Stones containing struvite, carbonate apatite, or ammonium urate were classified as infectious; all others were classified as non-infectious.

Stone components included calcium oxalate monohydrate in 302 patients (82.7%), carbonate apatite in 190 (52.1%), calcium oxalate dihydrate in 55 (15.1%), struvite in 37 (10.1%), and brushite in six (1.6%). Percentages exceeded 100% because most stones were mixed.

### 2.5 Outcome definitions and imaging assessment

The primary outcome was SFR 30 ± 7 days after surgery. Stone-free status was defined as no residual fragment ≥4 mm on KUB radiography. Patients receiving additional stone-directed treatment before assessment were classified as not stone-free. Two physicians blinded to treatment independently reviewed KUB images; disagreements were adjudicated by a blinded senior physician. Formal interobserver agreement was not calculated.

Recorded safety-related outcomes were postoperative fever, additional analgesic use, transfusion, and laboratory changes. Fever was any tympanic or axillary temperature ≥37.3 °C from surgery completion to discharge. Analgesic use was rescue medication beyond routine treatment, mainly parecoxib or tramadol. Transfusion was postoperative red-cell transfusion after hemoglobin decreased to <8 g/dL. Fasting blood was sampled on postoperative day 1; the latest preoperative value was baseline. ΔCRE, ΔCRP, ΔWBC, and ΔPCT were postoperative minus preoperative values, whereas ΔHB was preoperative minus postoperative hemoglobin.

Safety outcomes were retrospectively ascertained from available routine medical records. Medication-related events and postoperative ICU admissions were assessed during the index hospitalization, whereas readmissions and surgical, endoscopic, or radiological interventions were assessed through postoperative day 30. Clavien-Dindo grades were reconstructed using standard definitions through review of physician orders, progress notes, ICU transfer and hospitalization records, and operative or procedural records, with grading based on the most intensive treatment documented. ICU admission was defined as any documented postoperative ICU stay because planned and unplanned admissions could not be reliably distinguished. ICU admission alone was not considered sufficient for Clavien-Dindo grade IV; grade IV required a documented life-threatening complication with organ dysfunction requiring intermediate or intensive care.

### 2.6 Statistical analysis

Analyses were performed using R version 4.4.1. Continuous variables are reported as mean ± standard deviation or median (interquartile range), as appropriate, and categorical variables as *n* (%). Complete-case analyses were performed without imputation.

Two independent 1:1 propensity score-matched cohorts were constructed for Standard PCNL vs. NS and Standard PCNL vs. NR. Propensity scores were estimated using logistic regression incorporating age, sex, body mass index, diabetes, stone count, total stone diameter, stone density, parallel calices, Guy's stone score, and B.T.C.H. grade. Nearest-neighbor Mahalanobis-distance matching without replacement was performed within a caliper of 0.20 standard deviations of the logit propensity score. Patients outside common support were excluded, after which propensity scores were re-estimated. The estimand was the average treatment effect among successfully matched treated patients within common support. Covariate balance was assessed using absolute standardized mean differences (SMDs) based on pooled standard deviations, with multicategory variables represented by indicator variables. An absolute SMD <0.10 indicated adequate balance.

Binary outcomes were analyzed using two-sided exact McNemar tests, with discordant-pair counts retained. Treatment effects were reported as paired absolute risk differences with 95% confidence intervals (CIs). Paired Wald CIs were used except for the Standard PCNL–NR SFR comparison, for which a conditional exact CI was calculated. Continuous outcomes were analyzed using paired t-tests when within-pair differences were approximately normally distributed and Wilcoxon signed-rank tests otherwise. Effects were reported as mean paired differences or Hodges–Lehmann paired location shifts with 95% CIs, as appropriate.

The 1-month stone-free rate (SFR) was the primary outcome. Standard PCNL–NS and Standard PCNL–NR constituted the two principal treatment contrasts. *P* values from these two SFR comparisons were adjusted using the Holm method, and both unadjusted and Holm-adjusted *P* values were reported. All other perioperative outcomes and subgroup analyses were considered exploratory, without multiplicity adjustment.

Effect modification by infectious stone status was explored in all matched patients using a linear probability model containing treatment, infectious stone status, and their interaction, with matched-pair-clustered HC1 variance estimates. Cluster-robust logistic regression and exact conditional logistic regression stratified by matched pair were performed as sensitivity analyses. An additional supportive analysis retained only pairs concordant for infectious stone status.

A restrictive infection-stone sensitivity analysis was not feasible because quantitative stone-composition data and complete microbiological data were unavailable. Unless otherwise specified, all tests were two-sided, with *P* < 0.05 indicating statistical significance.

The study was reported in accordance with the STROBE guidelines, and the completed STROBE checklist is provided as Supplementary Material.

## 3 Results

### 3.1 Baseline characteristics after propensity score matching

Matching produced 48 Standard PCNL–NS and 86 Standard PCNL–NR pairs. Common-support intervals were 0.026–0.802 and 0.095–0.759, respectively. Six Standard PCNL and 13 NS patients were outside support in the first comparison, and six Standard PCNL patients and no NR patients in the second. Sixteen Standard PCNL controls appeared in both independent comparisons. All matched covariates had absolute SMDs <0.10, and overlap improved (Tables 1, 2; Figure 1).

**Table 1:** Baseline characteristics of standard PCNL and NS after PSM.

Variable	PCNL (*n* = 48)	NS (*n* = 48)	*P value*	SMD
Age (years)	51.3 ± 18.9	52.6 ± 14.1	0.665	0.074
Sex, *n* (%)			0.839	0.084
Male	26 (54.2)	28 (58.3)		
Female	22 (45.8)	20 (41.7)		
BMI (kg/m²)	24.7 ± 4.2	24.9 ± 4.4	0.872	0.028
Diabetes, *n* (%)	6 (12.5)	7 (14.6)	1.000	0.061
Stone count	2.0 (1.0–2.0)	2.0 (1.0–3.0)	0.695	0.066
Total stone diameter (mm)	33.5 (27.8–39.0)	33.1 (27.2–37.1)	0.691	0.007
Stone density (HU)	972.4 ± 319.4	964.9 ± 320.0	0.897	0.023
B.T.C.H. classification, *n* (%)			1.000	0.042
I	24 (50.0)	23 (47.9)		
II	24 (50.0)	25 (52.1)		
Guy's stone score, *n* (%)			1.000	0.000
II	14 (29.2)	14 (29.2)		
III	7 (14.6)	7 (14.6)		
IV	27 (56.2)	27 (56.2)		
Parallel calices, *n* (%)			1.000	0.044
Yes	16 (33.3)	17 (35.4)		
No	32 (66.7)	31 (64.6)		

**Table 2:** Baseline characteristics of standard PCNL and NR after PSM.

Variable	PCNL (*n* = 86)	NR (*n* = 86)	*P* value	SMD
Age (years)	52.0 ± 13.6	53.3 ± 13.4	0.467	0.091
Sex, *n* (%)			1.000	0.025
Male	60 (69.8)	61 (70.9)		
Female	26 (30.2)	25 (29.1)		
BMI (kg/m²)	25.6 ± 3.4	25.9 ± 3.8	0.505	0.074
Diabetes, *n* (%)	14 (16.3)	17 (19.8)	0.607	0.091
Stone count	2.0 (1.0–3.0)	2.0 (1.0–2.0)	0.495	0.051
Total stone diameter (mm)	25.9 (22.0–30.0)	26.1 (21.1–31.0)	0.915	0.041
Stone density (HU)	906.0 ± 272.8	885.7 ± 304.7	0.520	0.070
B.T.C.H. classification, *n* (%)			0.824	0.048
I	53 (61.6)	55 (64.0)		
II	33 (38.4)	31 (36.0)		
Guy's stone score, *n* (%)			0.462	0.033
II	22 (25.6)	22 (25.6)		
III	12 (14.0)	13 (15.1)		
IV	52 (60.5)	51 (59.3)		
Parallel calices, *n* (%)			1.000	0.000
Yes	25 (29.1)	25 (29.1)		
No	61 (70.9)	61 (70.9)		

**Figure 1:**
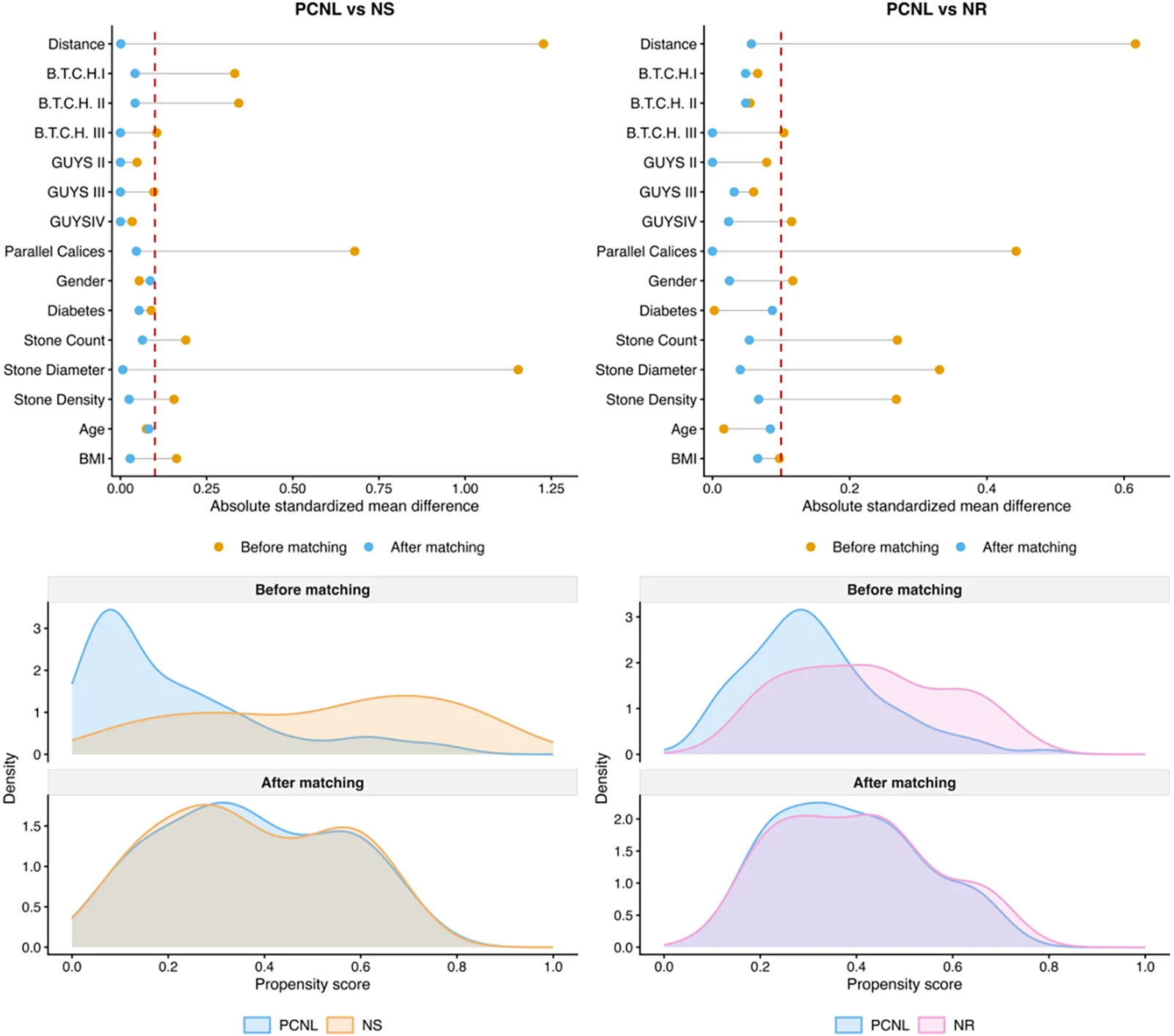
Covariate balance and propensity-score overlap before and after propensity score matching. Distance denotes the propensity-score distance used for matching. The red dashed line indicates an absolute standardized mean difference of 0.10. The upper panels show absolute SMDs before and after matching, and the lower panels show propensity-score distributions. The Standard PCNL–NS and Standard PCNL–NR comparisons represent independent matched cohorts.

Of 490 patients, 365 were eligible after 125 exclusions; none was excluded for incomplete data (Figure 2). The 32 radiolucent stone exclusions comprised 21 Standard PCNL, three NS, and eight NR cases. GSS grade I was absent after matching because Needle-perc was mainly selected for multiple or anatomically complex stones and such cases were uncommon in the NS and NR groups. Preoperative urine-culture results were summarized separately for the two independent matched cohorts. In the Standard PCNL–NS cohort, culture results were available for 92/96 patients, of whom 33/92 (35.9%) had positive cultures. Positive cultures were identified in 20/46 (43.5%) evaluable Standard PCNL patients and 13/46 (28.3%) evaluable NS patients; four culture records were unavailable. In the Standard PCNL–NR cohort, culture results were available for 164/172 patients, with positive cultures in 57/164 (34.8%). The corresponding proportions were 30/81 (37.0%) in the Standard PCNL group and 27/83 (32.5%) in the NR group; eight culture records were unavailable.

**Figure 2:**
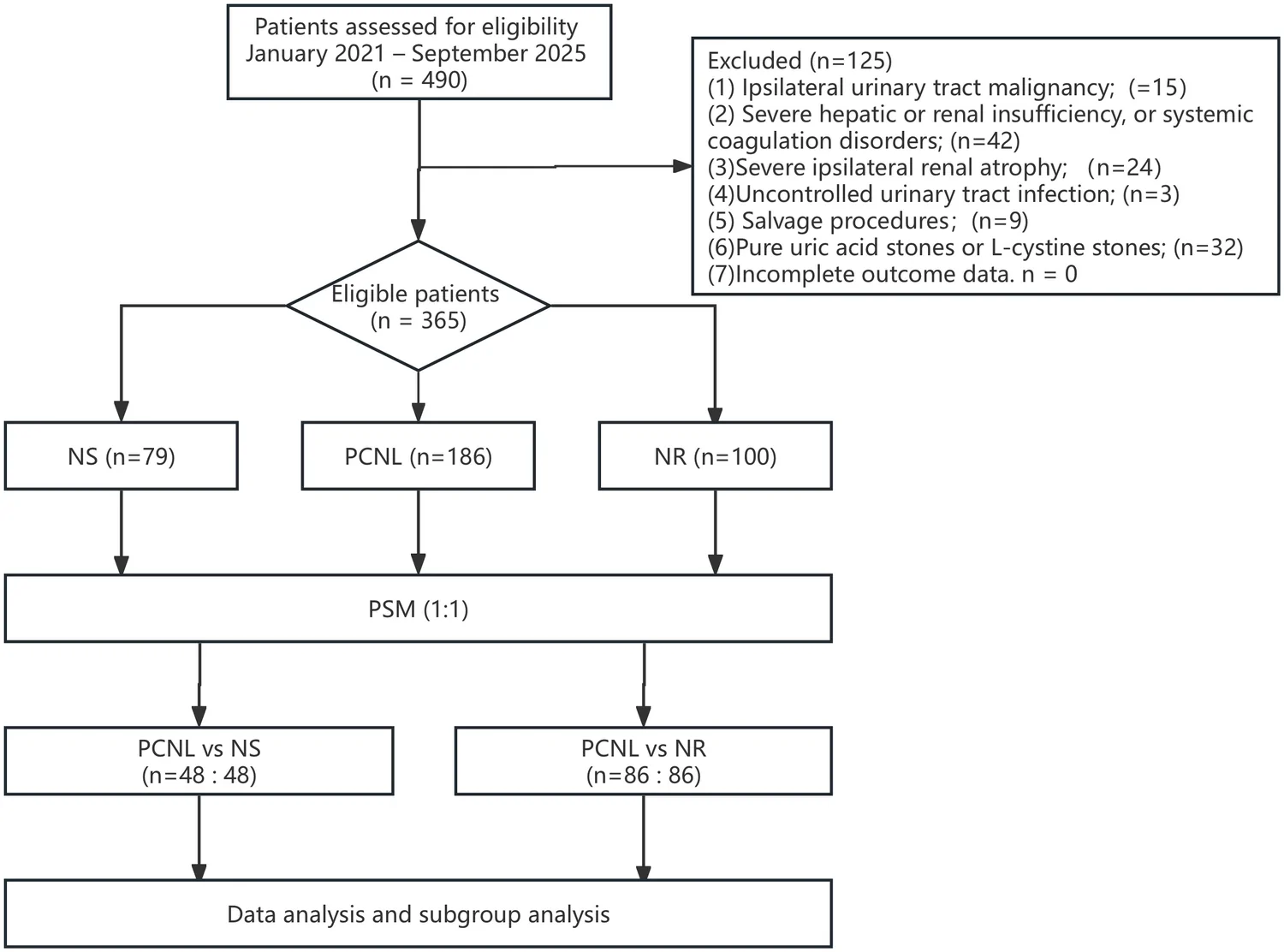
Flow chart.

*Escherichia coli* and *Enterococcus faecalis* were the predominant organisms in both matched cohorts. In the Standard PCNL–NS cohort, they accounted for 11 and 8 isolates, respectively; in the Standard PCNL–NR cohort, they accounted for 16 and 11 isolates, respectively. Other cultured organisms included *Klebsiella pneumoniae*, *Proteus mirabilis*, *Streptococcus agalactiae*, and several less frequent isolates. Polymicrobial cultures were identified in two patients in the Standard PCNL–NS cohort and four patients in the Standard PCNL–NR cohort.

ASA physical-status classifications were predominantly II or III in both matched cohorts. In the Standard PCNL–NS cohort, 36/48 (75.0%) Standard PCNL patients were classified as ASA II and 12/48 (25.0%) as ASA III. In the NS group, 34/48 (70.8%) patients were classified as ASA II and 13/48 (27.1%) as ASA III, with one missing record. In the Standard PCNL–NR cohort, the Standard PCNL group included 62/86 (72.1%) ASA II and 23/86 (26.7%) ASA III patients, with one missing record. The NR group included 2/86 (2.3%) ASA I, 65/86 (75.6%) ASA II, and 19/86 (22.1%) ASA III patients.

In the Standard PCNL–NS cohort, the recorded Clavien–Dindo classifications comprised one patient without a recorded complication, 34 grade I patients, and 13 grade II patients in the Standard PCNL group, compared with 34 grade I and 13 grade II patients in the NS group; one NS record was unavailable. In the Standard PCNL–NR cohort, the Standard PCNL group comprised one patient without a recorded complication, 58 grade I, 25 grade II, and one grade IIIa patient, with one missing record, whereas the NR group comprised 63 grade I and 23 grade II patients. One ICU admission was recorded in the Standard PCNL group in each matched comparison, whereas no ICU admissions occurred in the NS or NR groups. Because the matched cohorts were constructed independently and could include overlapping Standard PCNL controls, ICU events were not pooled across comparisons.

Seven individual surgeons were recorded as primary operators in the matched datasets. In the Standard PCNL–NS cohort, seven surgeons were represented in the Standard PCNL group and five in the NS group, with one missing NS surgeon record. In the Standard PCNL–NR cohort, seven surgeons were represented in each group, with one missing Standard PCNL surgeon record. The most frequently recorded surgeon performed 17/47 (36.2%) NS procedures with available surgeon data and 43/86 (50.0%) NR procedures.

The calendar-year distributions differed between treatment groups. In the Standard PCNL–NS cohort, 44/48 (91.7%) NS procedures were performed during 2024–2025, compared with 12/48 (25.0%) matched Standard PCNL procedures. In the Standard PCNL–NR cohort, 45/86 (52.3%) NR procedures and 59/86 (68.6%) matched Standard PCNL procedures were performed during 2024–2025. After harmonization of the calendar-year coding, the NR procedures were distributed across 2021 (16/86, 18.6%), 2022 (19/86, 22.1%), 2023 (6/86, 7.0%), 2024 (9/86, 10.5%), and 2025 (36/86, 41.9%).

Characteristics of matched and unmatched Standard PCNL patients are presented in Supplementary Tables 1, 2. In the Standard PCNL–NS analysis, matched Standard PCNL patients had a larger median stone diameter (33.5 vs. 25.6 mm; SMD = 1.046) and a higher prevalence of parallel calices (33.3% vs. 14.5%; SMD = 0.453) than unmatched patients. In the Standard PCNL–NR analysis, matched patients had a smaller median stone diameter (25.9 vs. 28.9 mm; SMD = 0.442), lower mean stone density (906.0 vs. 997.6 HU; SMD = 0.315), and a higher prevalence of parallel calices (29.1% vs. 11.0%; SMD = 0.463). These findings indicate that the two matched comparisons represented distinct subsets of the original Standard PCNL population.

### 3.2 Overall outcomes after propensity score matching

In the Standard PCNL–NS cohort, SFR was 72.9% vs. 85.4% (35/48 vs. 41/48; paired RD, 12.5 percentage points; 95% CI, −3.6 to 28.6; exact McNemar *P* = 0.210; Holm-adjusted *P* = 0.210).

NS was associated with less fever (8.3% vs. 35.4%; RD, −27.1 percentage points; 95% CI, −43.3 to −10.8; *P* = 0.004), more analgesic use (29.2% vs. 8.3%; RD, 20.8; 95% CI, 7.8 to 33.8; *P* = 0.006), and a smaller WBC increase (Hodges–Lehmann shift, −1.37 × 10^9^/L; 95% CI, −2.69 to −0.20; *P* = 0.024). Transfusion occurred in three NS patients and none after Standard PCNL (*P* = 0.250); other outcomes did not differ statistically (Table 3).

**Table 3:** Outcomes after PSM.

Outcome	PCNL vs. NS					PCNL vs. NR				
	PCNL (*n* = 48)	NS (*n* = 48)	Effect estimate (95% CI)	Test	*P* value	PCNL (*n* = 86)	NR (*n* = 86)	Effect estimate (95% CI)	Test	*P* value
Stone-free rate, n/N (%)	35/48 (72.9%)	41/48 (85.4%)	RD 12.5% (−3.6 to 28.6)	Exact McNemar	0.210	69/86 (80.2%)	79/86 (91.9%)	RD 11.6% (−0.1% to 20.1%)	Exact McNemar	0.052
Analgesic use, n/N (%)	4/48 (8.3%)	14/48 (29.2%)	RD 20.8% (7.8 to 33.8)	Exact McNemar	0.006	20/86 (23.3%)	8/86 (9.3%)	RD −14.0% (−25.3 to −2.6)	Exact McNemar	0.029
Blood transfusion, n/N (%)	0/48 (0.0%)	3/48 (6.2%)	RD 6.2% (−0.7 to 13.2)	Exact McNemar	0.250	1/86 (1.2%)	1/86 (1.2%)	RD 0.0% (−3.2 to 3.2)	Exact McNemar	1.000
Postoperative fever, n/N (%)	17/48 (35.4%)	4/48 (8.3%)	RD −27.1% (−43.3 to −10.8)	Exact McNemar	0.004	21/86 (24.4%)	19/86 (22.1%)	RD −2.3% (−14.4 to 9.8)	Exact McNemar	0.851
Operative time (min)	108.0 ± 37.6	116.8 ± 39.6	MD 8.8 (−6.3 to 23.8)	Paired t-test	0.247	89.0 (72.0–119.2)	120.0 (99.2–146.8)	HL 31.0 (19.0 to 41.0)	Wilcoxon	<0.001
Postoperative hospital stay (days)	6.0 (4.0–6.0)	5.0 (4.8–7.0)	HL 0.0 (−1.0 to 1.0)	Wilcoxon	0.894	5.0 (5.0–6.0)	3.0 (2.0–5.0)	HL −2.5 (−3.0 to −2.0)	Wilcoxon	<0.001
ΔCRE	5.40 ± 14.86	5.36 ± 12.13	MD −0.04 (−5.35 to 5.27)	Paired t-test	0.988	4.50 (−2.00–10.00)	3.50 (−1.00–12.00)	HL −1.00 (−5.50 to 4.00)	Wilcoxon	0.611
ΔCRP	2.38 (0.15–10.31)	1.61 (0.38–7.01)	HL −1.79 (−7.37 to 3.09)	Wilcoxon	0.430	3.48 (1.38–10.78)	3.60 (1.43–9.32)	HL 0.10 (−2.64 to 2.92)	Wilcoxon	0.950
ΔHB	6.69 ± 8.60	5.27 ± 5.52	MD −1.42 (−4.74 to 1.91)	Paired t-test	0.396	7.57 ± 9.92	6.90 ± 7.36	MD −0.67 (−3.40 to 2.05)	Paired t-test	0.624
ΔWBC	3.83 (2.36–5.38)	2.74 (1.15–3.70)	HL −1.37 (−2.69 to −0.20)	Wilcoxon	0.024	3.56 ± 3.67	4.60 ± 3.20	MD 1.04 (−0.05 to 2.13)	Paired t-test	0.061
ΔPCT	0.01 (−0.01–0.05)	0.00 (0.00–0.04)	HL 0.00 (−0.03 to 0.03)	Wilcoxon	0.894	0.01 (−0.01–0.05)	0.01 (−0.01–0.03)	HL 0.00 (−0.02 to 0.01)	Wilcoxon	0.728

Values are *n*/*N* (%), mean ± SD, or median (IQR). Effect estimates are treatment minus PCNL. RD, risk difference; MD, mean paired difference; HL, Hodges–Lehmann paired location shift. *P* values are from two-sided exact McNemar tests, paired t-tests, or Wilcoxon signed-rank tests, as shown.

In the Standard PCNL–NR cohort, SFR was 80.2% vs. 91.9% (69/86 vs. 79/86; paired RD, 11.6 percentage points; conditional exact 95% CI, −0.1 to 20.1; exact McNemar *P* = 0.052; Holm-adjusted *P* = 0.104). NR was associated with less analgesic use (9.3% vs. 23.3%; RD, −14.0; 95% CI, −25.3 to −2.6; *P* = 0.029), longer operative time (120.0 vs. 89.0 min; Hodges–Lehmann shift, 31.0; 95% CI, 19.0 to 41.0; *P* < 0.001), and shorter hospitalization (3.0 vs. 5.0 days; shift, −2.5; 95% CI, −3.0 to −2.0; *P* < 0.001). Other outcomes did not differ statistically (Table 3). Clavien-Dindo grades and postoperative ICU admissions are reported numerically in Supplementary Table 3, with outcome-specific denominators and missing observations shown separately. Because the two matched cohorts were constructed independently and could contain overlapping Standard PCNL controls, ICU events were not pooled across comparisons.

### 3.3 Exploratory analysis according to infectious stone status

For Standard PCNL–NS, SFR RDs favoring NS were 9.1 percentage points in non-infectious stones (95% CI, −16.7 to 34.8) and 17.1 in infectious stones (95% CI, −6.3 to 40.5); the interaction was 8.0 (95% CI, −26.9 to 42.9; *P* = 0.647) (Table 4).

**Table 4:** Supportive outcome analyses of standard PCNL versus NS in infection-status-concordant matched pairs.

Outcome	Infectious stones (11 matched pairs)					Non-infectious stones (11 matched pairs)				
	Standard PCNL	NS	Paired effect estimate (95% CI)	Test/statistic	*P* value	Standard PCNL	NS	Paired effect estimate (95% CI)	Test/statistic	*P* value
Analgesic	2/11 (18.2%)	2/11 (18.2%)	0.0 (−26.4 to 26.4)*	Exact McNemar	1.000	1/11 (9.1%)	3/11 (27.3%)	18.2 (−5.7 to 42.1)*	Exact McNemar	0.500
Blood transfusion	0/11 (0.0%)	1/11 (9.1%)	9.1 (−8.7 to 26.9)*	Exact McNemar	1.000	0/11 (0.0%)	0/11 (0.0%)	0.0 (0.0 to 0.0)*	Exact McNemar	1.000
Postoperative fever	4/11 (36.4%)	2/11 (18.2%)	−18.2 (−62.5 to 26.2)*	Exact McNemar	0.688	5/11 (45.5%)	0/11 (0.0%)	−45.5 (−76.3 to −14.6)*	Exact McNemar	0.063
Operative time (min)	106.00 ± 31.87	108.36 ± 30.35	2.364 (−20.013 to 24.741)^†^	*t* = 0.235	0.819	104.09 ± 34.59	113.82 ± 30.17	9.727 (−23.249 to 42.704)^†^	*t* = 0.657	0.526
Postoperative hospital stay (days)	5.45 ± 1.81	5.55 ± 2.30	0.091 (−1.843 to 2.025)^†^	*t* = 0.105	0.919	6.27 ± 2.45	6.27 ± 2.72	0.000 (−2.308 to 2.308)^†^	*t* = 0.000	1.000
ΔCRE	2.00 (−1.00–10.50)	5.00 (5.00–8.00)	3.000 (−4.000 to 15.000)^‡^	*V* = 40.500	0.504	5.00 (−1.80–16.50)	4.00 (−1.10–14.30)	3.582 (−11.000 to 14.600)^‡^	*V* = 40.000	0.533
ΔCRP	2.63 (0.65–19.21)	3.96 (1.18–7.54)	−2.718 (−52.455 to 14.640)^‡^	*V* = 29.000	0.722	9.49 ± 18.97	10.63 ± 17.49	1.137 (−14.636 to 16.910)^†^	*t* = 0.161	0.876
ΔHB	8.73 ± 8.38	5.09 ± 3.67	−3.636 (−10.227 to 2.955)^†^	*t* = −1.229	0.247	8.09 ± 7.98	7.09 ± 3.88	−1.000 (−8.493 to 6.493)^†^	*t* = −0.297	0.772
ΔWBC	3.66 (2.84–4.67)	2.82 (1.62–3.71)	−1.084 (−7.435 to 1.485)^‡^	*V* = 22.000	0.328	3.72 ± 2.21	2.02 ± 2.84	−1.700 (−3.637 to 0.237)^†^	*t* = −1.956	0.079
ΔPCT	0.00 (−0.02–0.04)	0.01 (0.00–0.06)	0.015 (−31.873 to 1.956)^‡^	*V* = 38.000	0.657	0.01 (−0.01–0.02)	−0.00 (−0.01–0.06)	0.031 (−0.018 to 8.574)^‡^	*V* = 50.000	0.131

Values are *n*/*N* (%), mean ± SD, or median (IQR), as appropriate.

* Paired absolute risk difference (NS minus Standard PCNL), in percentage points, with paired Wald 95% CI.

† Mean paired difference (NS minus Standard PCNL) with 95% CI.

‡ Hodges–Lehmann paired location shift (NS minus Standard PCNL) with 95% CI. *P* values were from two-sided exact McNemar, paired t, or Wilcoxon signed-rank tests, as appropriate.

For Standard PCNL–NR, SFR RDs were 11.3 percentage points in non-infectious stones (95% CI, −4.3 to 26.9) and 12.1 in infectious stones (95% CI, −2.1 to 26.2); the interaction was 0.8 (95% CI, −20.0 to 21.5; *P* = 0.942). Logistic and conditional logistic sensitivity analyses were consistent (Table 5, Figure 3).

**Table 5:** Supportive outcome analyses of standard PCNL versus NR in infection-status-concordant matched pairs.

Outcome	Infectious stones (18 matched pairs)					Non-infectious stones (18 matched pairs)				
	Standard PCNL	NR	Paired effect estimate (95% CI)	Test/statistic	*P* value	Standard PCNL	NR	Paired effect estimate (95% CI)	Test/statistic	*P* value
Analgesic	4/18 (22.2%)	2/18 (11.1%)	−11.1 (−38.0 to 15.8)*	Exact McNemar	0.688	6/18 (33.3%)	2/18 (11.1%)	−22.2 (−47.6 to 3.1)*	Exact McNemar	0.219
Blood transfusion	0/18 (0.0%)	0/18 (0.0%)	0.0 (0.0 to 0.0)*	Exact McNemar	1.000	0/18 (0.0%)	0/18 (0.0%)	0.0 (0.0 to 0.0)*	Exact McNemar	1.000
Postoperative fever	4/18 (22.2%)	3/18 (16.7%)	−5.6 (−35.1 to 24.0)*	Exact McNemar	1.000	3/18 (16.7%)	3/18 (16.7%)	0.0 (−27.4 to 27.4)*	Exact McNemar	1.000
Operative time (min)	108.39 ± 42.28	116.78 ± 51.57	8.389 (−32.031 to 48.809)^†^	*t* = 0.438	0.667	83.00 ± 24.25	121.94 ± 31.57	38.944 (19.473 to 58.416)^†^	*t* = 4.220	<0.001
Postoperative hospital stay (days)	5.39 ± 1.65	3.67 ± 2.33	−1.722 (−3.158 to −0.287)^†^	*t* = −2.532	0.021	6.00 (5.00–7.00)	3.00 (2.25–4.75)	−3.000 (−4.500 to −1.000)^‡^	*V* = 27.500	0.036
ΔCRE	5.23 ± 13.52	2.83 ± 14.75	−2.394 (−11.602 to 6.814)^†^	*t* = −0.549	0.590	9.78 ± 16.18	11.67 ± 21.87	1.889 (−12.814 to 16.591)^†^	*t* = 0.271	0.790
ΔCRP	3.42 (−0.39–9.34)	1.90 (0.69–3.39)	−2.100 (−15.055 to 3.150)^‡^	*V* = 67.000	0.420	4.77 (1.67–10.90)	6.56 (3.50–9.73)	0.765 (−6.635 to 6.360)^‡^	*V* = 92.000	0.777
ΔHB	6.89 ± 9.99	5.44 ± 5.44	−1.444 (−6.412 to 3.523)^†^	*t* = −0.614	0.548	10.22 ± 7.82	6.44 ± 8.91	−3.778 (−10.537 to 2.982)^†^	*t* = −1.179	0.255
ΔWBC	4.47 ± 4.90	4.07 ± 2.82	−0.401 (−3.168 to 2.367)^†^	*t* = −0.305	0.764	3.13 ± 3.39	4.26 ± 2.82	1.139 (−1.141 to 3.419)^†^	*t* = 1.054	0.307
ΔPCT	0.01 (−0.01–0.21)	0.01 (−0.01–0.02)	−0.069 (−2.115 to 0.009)^‡^	*V* = 51.000	0.133	0.02 (0.00–0.03)	0.01 (−0.01–0.04)	0.006 (−0.019 to 0.053)^‡^	*V* = 98.000	0.586

Values are *n*/*N* (%), mean ± SD, or median (IQR), as appropriate.

* Paired absolute risk difference (NR minus Standard PCNL), in percentage points, with paired Wald 95% CI.

† Mean paired difference (NR minus Standard PCNL) with 95% CI.

‡ Hodges–Lehmann paired location shift (NR minus Standard PCNL) with 95% CI. *P* values were from two-sided exact McNemar, paired *t*, or Wilcoxon signed-rank tests, as appropriate.

**Figure 3:**
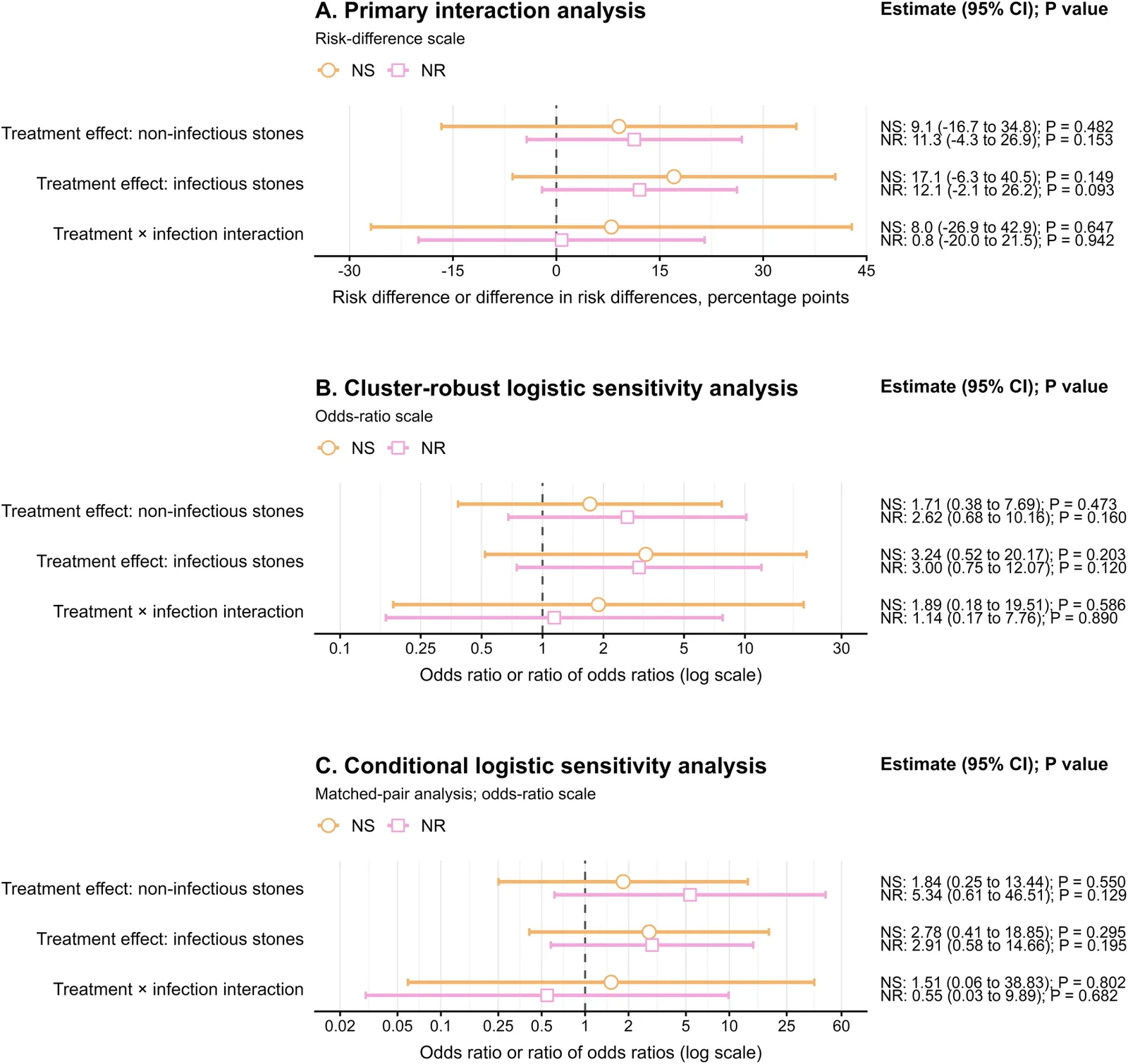
Treatment-by-infectious-stone-status interaction analyses for the 1-month stone-free rate. **(A)** Risk differences from primary linear probability models with matched-pair-clustered robust variance. **(B)** Odds ratios from cluster-robust logistic models. **(C)** Odds ratios from exact conditional logistic models stratified by matched pair. Points and lines indicate estimates and 95% confidence intervals.

Concordant-pair analyses retained 11 infectious and 11 non-infectious NS pairs and 18 pairs per NR stratum, excluding 26 and 50 pairs, respectively. Estimates were imprecise; NR was associated with shorter hospitalization in both strata and longer operative time in the non-infectious stratum. These reduced-sample analyses were supportive only.

## 4 Discussion

Both NS and NR had numerically higher 1-month SFRs than their matched Standard PCNL controls, but neither established superiority after paired testing and Holm adjustment. NS was associated with less fever and a smaller WBC increase but greater analgesic use; NR with less analgesic use and shorter hospitalization but longer operative time. Secondary findings were exploratory.

PCNL remains first-line treatment for stones >2 cm (3), although access-related morbidity has encouraged miniaturized techniques (13, 14). Flexible and navigable suction ureteral access sheath systems have expanded RIRS for larger stones (15), but unfavorable calyces, narrow infundibula, and limited ureteroscopic reach remain challenging (16, 17). Needle-perc provides an undilated 4.2-Fr tract under ultrasound guidance with visual confirmation (5, 18). NS and NR were therefore evaluated separately against Standard PCNL.

Comparisons between matched and unmatched Standard PCNL patients further clarify the populations represented by the two analyses. In the Standard PCNL–NS analysis, matched Standard PCNL patients had larger stones and a higher prevalence of parallel calices than unmatched patients. In the Standard PCNL–NR analysis, matched patients also more frequently had parallel calices, although their stone diameter and density were lower. Thus, the matching procedures selected different subsets of the Standard PCNL population, particularly patients with anatomical characteristics relevant to calyceal accessibility. In these selected populations, the numerically higher SFRs observed with Needle-perc-assisted surgery may partly reflect the ability of the small-caliber tract to provide targeted access to parallel or otherwise unfavorable calices and facilitate the management of residual fragments. However, comparisons between matched and unmatched controls describe selection into the matched cohorts and do not themselves establish the mechanism of treatment benefit or treatment-effect heterogeneity. Accordingly, the present estimates are primarily applicable to successfully matched patients within common support rather than to the entire Standard PCNL population.

Within this context, the higher SFR estimate with NS may reflect targeted access to residual stones in parallel or unfavorable calices without dilating an additional conventional tract, while facilitating fragment movement toward the main extraction pathway. The lower fever frequency and smaller WBC increase may suggest a reduced inflammatory response; however, NS was also associated with greater analgesic use, and three transfusions occurred in the NS group compared with none in its matched Standard PCNL group. These exploratory findings should therefore be interpreted cautiously. NR provides antegrade access when RIRS alone cannot reach or treat the stone. Its SFR estimate favored NR without establishing superiority. Less analgesic use and shorter hospitalization were accompanied by longer operative time, likely reflecting dual-pathway coordination and puncture. Hospitalization may also have been shortened by the planned tubeless pathway, whereas Standard PCNL routinely used nephrostomy drainage.

Any stone containing struvite, carbonate apatite, or ammonium urate was classified as infectious. This broad definition may misclassify mixed stones containing only minor carbonate apatite. Quantitative proportions and complete microbiological data were unavailable, precluding a restrictive definition. No treatment-by-infection interaction was statistically significant, but wide CIs did not exclude meaningful heterogeneity. Concordant-pair strata retained fewer than half of original pairs and showed residual imbalance, including absolute SMDs >0.50; these analyses were supportive only.

Needle-perc should not replace Standard PCNL universally. NS may supplement an inaccessible PCNL tract, whereas NR may be considered after ureteroscopy confirms that RIRS alone is insufficient. Selection should reflect stone burden, anatomy, retrograde access, and intraoperative findings.

This study has several limitations. Its single-center retrospective design, convenience sample, and lack of an *a priori* sample-size calculation limited precision, particularly for rare events and subgroup interactions. Despite PSM, selection bias and residual confounding remained, and the two comparisons involved distinct Standard PCNL control populations. Treatment selection, exclusion of salvage conversions, and unadjusted surgeon, calendar-year, and learning-curve effects may also have influenced the findings. The broad infection-stone definition and incomplete compositional, microbiological, antimicrobial-treatment, and drainage data may have caused misclassification; renal-pelvic urine and stone cultures were not routinely obtained. KUB is less sensitive than NCCT for detecting residual fragments. Although images were independently assessed by two physicians with adjudication of disagreements, interobserver agreement could not be reliably reconstructed. Retrospective Clavien–Dindo assessment may also have missed complications; therefore, nonsignificant findings do not establish equivalent safety. Finally, the shorter hospitalization after NR may partly reflect its tubeless postoperative pathway.

## 5 Conclusion

SFR estimates numerically favored NS and NR without establishing superiority. NR was associated with longer operative time and shorter hospitalization, partly attributable to its tubeless pathway. No infection-status interaction was detected, but estimates were imprecise and subgroup imbalance remained. Prospective studies with standardized NCCT and adequate power are needed.

## Data Availability

The datasets generated and/or analyzed during the current study are not publicly available because they contain potentially identifiable clinical information but are available from the corresponding author on reasonable request and subject to institutional and ethical approval. Requests to access the datasets should be directed to Jianxing Li, lijianxing2015@163.com.
