# Supplementary material for: Clinical outcomes of needle-perc-assisted endoscopic surgery for 2–4 cm renal calculi: a propensity score-matched comparative study with subgroup analysis by stone infection status
Source: Front Med (Lausanne). 2026 Oct 22;13:1938484. doi: 10.3389/fmed.2026.1938484 (PMC13738237; doi:10.3389/fmed.2026.1938484)
Supplement: Supplementary file 1 [file Table1.docx]

**Supplementary Table 1. Characteristics of Matched and Unmatched Standard PCNL Patients in the Standard PCNL-NS Analysis**

| **Variable** | **Matched Standard PCNL (n = 48)** | **Unmatched Standard PCNL (n = 138)** | ***P value*** | **SMD** |
| --- | --- | --- | --- | --- |
| **Age (years)** | 51.3 ± 18.9 | 53.3 ± 14.8 | 0.513 | 0.117 |
| **Gender, n (%)** |  |  | 0.114 | 0.289 |
| Male | 26 (54.2) | 94 (68.1) |  |  |
| Female | 22 (45.8) | 44 (31.9) |  |  |
| **BMI (kg/m²)** | 24.7 ± 4.2 | 25.8 ± 4.3 | 0.130 | 0.254 |
| **Diabetes, n (%)** | 6 (12.5) | 31 (22.5) | 0.149 | 0.265 |
| **Stone count** | 2.0 (1.0–2.0) | 1.0 (1.0–2.0) | 0.068 | 0.205 |
| **Stone diameter (mm)** | 33.5 (27.8–39.0) | 25.6 (22.0–30.0) | <0.001 | 1.046 |
| **Stone density (HU)** | 972.4 ± 319.4 | 949.2 ± 287.4 | 0.658 | 0.076 |
| **BTCHC, n (%)** |  |  | 0.085 | 0.359 |
| I | 24 (50.0) | 91 (65.9) |  |  |
| II | 24 (50.0) | 46 (33.3) |  |  |
| III | 0 (0.0) | 1 (0.7) |  |  |
| **Guy’s stone score, n (%)** |  |  | 0.874 | 0.096 |
| II | 14 (29.2) | 39 (28.3) |  |  |
| III | 7 (14.6) | 25 (18.1) |  |  |
| IV | 27 (56.2) | 74 (53.6) |  |  |
| **Parallel calices, n (%)** |  |  | 0.010 | 0.453 |
| Yes | 16 (33.3) | 20 (14.5) |  |  |
| No | 32 (66.7) | 118 (85.5) |  |  |

Data are presented as mean ± standard deviation, median (interquartile range), or n (%). P values were calculated using Welch’s t test for age, BMI, and stone density; the Wilcoxon rank-sum test for stone count and stone diameter; and two-sided Fisher’s exact test for categorical variables. SMDs are absolute standardized mean differences; generalized SMDs are reported for multilevel categorical variables.
