# Supplementary material for: Clinical outcomes of needle-perc-assisted endoscopic surgery for 2–4 cm renal calculi: a propensity score-matched comparative study with subgroup analysis by stone infection status
Source: Front Med (Lausanne). 2026 Oct 22;13:1938484. doi: 10.3389/fmed.2026.1938484 (PMC13738237; doi:10.3389/fmed.2026.1938484)
Supplement: Supplementary file 2 [file Table2.docx]

**Supplementary Table 2. Characteristics of Matched and Unmatched Standard PCNL Patients in the Standard PCNL–NR Analysis**

| **Variable** | **Matched Standard PCNL (n = 86)** | **Unmatched Standard PCNL (n = 100)** | ***P value*** | **SMD** |
| --- | --- | --- | --- | --- |
| **Age (years)** | 52.0 ± 13.6 | 53.5 ± 17.7 | 0.536 | 0.090 |
| **Gender, n (%)** |  |  | 0.171 | 0.206 |
| Male | 60 (69.8) | 60 (60.0) |  |  |
| Female | 26 (30.2) | 40 (40.0) |  |  |
| **BMI (kg/m²)** | 25.6 ± 3.4 | 25.5 ± 4.9 | 0.851 | 0.027 |
| **Diabetes, n (%)** | 14 (16.3) | 23 (23.0) | 0.274 | 0.170 |
| **Stone count** | 2.0 (1.0–3.0) | 1.0 (1.0–2.0) | 0.098 | 0.294 |
| **Stone diameter (mm)** | 25.9 (22.0–30.0) | 28.9 (24.2–34.2) | 0.005 | 0.442 |
| **Stone density (HU)** | 906.0 ± 272.8 | 997.6 ± 308.4 | 0.033 | 0.315 |
| **BTCHC, n (%)** |  |  | 1.000 | 0.144 |
| I | 53 (61.6) | 62 (62.0) |  |  |
| II | 33 (38.4) | 37 (37.0) |  |  |
| III | 0 (0.0) | 1 (1.0) |  |  |
| **Guy’s stone score, n (%)** |  |  | 0.279 | 0.238 |
| II | 22 (25.6) | 31 (31.0) |  |  |
| III | 12 (14.0) | 20 (20.0) |  |  |
| IV | 52 (60.5) | 49 (49.0) |  |  |
| **Parallel calices, n (%)** |  |  | 0.003 | 0.463 |
| Yes | 25 (29.1) | 11 (11.0) |  |  |
| No | 61 (70.9) | 89 (89.0) |  |  |

Data are presented as mean ± standard deviation, median (interquartile range), or n (%). P values were calculated using Welch’s t test for age, BMI, and stone density; the Wilcoxon rank-sum test for stone count and stone diameter; and two-sided Fisher’s exact test for categorical variables. SMDs are absolute standardized mean differences; generalized SMDs are reported for multilevel categorical variables.
