# Supplementary material for: Clinical outcomes of needle-perc-assisted endoscopic surgery for 2–4 cm renal calculi: a propensity score-matched comparative study with subgroup analysis by stone infection status
Source: Front Med (Lausanne). 2026 Oct 22;13:1938484. doi: 10.3389/fmed.2026.1938484 (PMC13738237; doi:10.3389/fmed.2026.1938484)
Supplement: Supplementary file 3 [file Table3.docx]

**Supplementary Table 3. Incompletely ascertained routine-record safety outcomes in the propensity score-matched cohorts**

| **Outcome** | **PCNL, n/N (%)** | | **Comparator, n/N (%)** |
| --- | --- | --- | --- |
| **Panel A. PCNL versus NS matched cohort** | |  |  |
| **Clavien-Dindo grade** |  | |  |
| No documented complication (grade 0) | 1/48 (2.1) | | 0/47 (0.0) |
| Grade I | 34/48 (70.8) | | 34/47 (72.3) |
| Grade II | 13/48 (27.1) | | 13/47 (27.7) |
| Grade IIIa | 0/48 (0.0) | | 0/47 (0.0) |
| Grade IIIb | 0/48 (0.0) | | 0/47 (0.0) |
| Grade IVa | 0/48 (0.0) | | 0/47 (0.0) |
| Grade IVb | 0/48 (0.0) | | 0/47 (0.0) |
| Grade V | 0/48 (0.0) | | 0/47 (0.0) |
| **Any documented postoperative ICU admission** | 1/48 (2.1) | | 0/47 (0.0) |
| Missing data, n | 0 | | 1 |
| **Panel B. PCNL versus NR matched cohort** | |  |  |
| **Clavien-Dindo grade** |  | |  |
| No documented complication (grade 0) | 1/85 (1.2) | | 0/86 (0.0) |
| Grade I | 58/85 (68.2) | | 63/86 (73.3) |
| Grade II | 25/85 (29.4) | | 23/86 (26.7) |
| Grade IIIa | 1/85 (1.2) | | 0/86 (0.0) |
| Grade IIIb | 0/85 (0.0) | | 0/86 (0.0) |
| Grade IVa | 0/85 (0.0) | | 0/86 (0.0) |
| Grade IVb | 0/85 (0.0) | | 0/86 (0.0) |
| Grade V | 0/85 (0.0) | | 0/86 (0.0) |
| **Any documented postoperative ICU admission** | 1/85 (1.2) | | 0/86 (0.0) |
| Missing data, n | 1 | | 0 |

Data are presented as n/N (%), with missing observations excluded and reported separately. For multiple complications, the highest Clavien–Dindo grade was used. Readmissions and interventions were assessed within 30 days. ICU admission referred to any documented postoperative ICU stay and alone did not constitute grade IV.
